# Optimization of Anterior Incision Placement for Distal Biceps Repair

**DOI:** 10.7759/cureus.3141

**Published:** 2018-08-14

**Authors:** Nikolai Klebanov, David H Wei, Brendan J Harrison, Hervey L Kimball

**Affiliations:** 1 Dermatology, Massachusetts General Hospital, Boston, USA; 2 Orthopaedic Surgery, Greenwich Hospital, Greenwich, USA; 3 Internal Medicine, Steward Carney Hospital/Tufts University School of Medicine, Dorchester, USA; 4 Orthopedic Surgery, New England Baptist Hospital, Boston, USA

**Keywords:** biceps tendon, biceps tendon rupture, radiographic anatomy

## Abstract

Introduction

Damage to the posterior interosseous nerve (PIN) is a known complication when using a cortical button during distal biceps tendon repair. Prior studies show that the trajectory of the drill through the biceps tuberosity can affect the distance from the PIN. We develop a mathematical model to predict the location of the tuberosity based on a palpable bony landmark and patient demographic factors.

Methods

The medical charts and elbow radiographs of (n = 82) adult patients were retrospectively reviewed. Using standard radiographic software, two observers measured the distance from the olecranon tip to the center of the biceps tuberosity. Multivariate regression analysis was used to build a linear model. The model was cross-validated with five arms from three distinct cadavers. A surgical wire was guided into the volar aspect of each forearm using the model, and a dissection was then performed to assess the proximity of the surgical wire to the insertion of the biceps tendon on the radial tuberosity.

Results

Olecranon-tuberosity distance (OTD) ranged from 52.3 mm to 77.2 mm (mean 66.5 mm). Univariate analyses revealed males had significantly longer OTD (mean 69.3 mm) compared to females (mean 61.2 mm, t-test, p < 0.001). Increased body mass index (BMI) weakly correlated with increased distance (Pearson’s r = 0.22, p = 0.048). Height showed strong positive correlation with increased distance (r = 0.77, p < 0.001). Multivariate regression revealed that significant predictive factors for olecranon-tuberosity distance were height (coefficient = 35.8, p < 0.001), BMI (coefficient = 0.14, p = 0.032), and male sex (coefficient = 3.17, p = 0.0039). The average error in the cadaveric validation, measured as distance from the surgical wire to the distal biceps insertion was 1.8 mm.

Conclusion

A highly accurate mathematical model can be used to predict the location of the biceps tuberosity in relation to the palpable tip of the olecranon, based only on height, BMI, and sex of the patient. Knowledge of this distance can guide accurate placement of the skin incision when a transverse single-incision approach is utilized for repair of the distal biceps tendon using a cortical button. Diagnostics showed the model to be less accurate near the extremes of the measurement. Since patients with a target incision point far removed from average would most benefit from such a model, we will continue by identifying and enrolling patients at the low and high ends of the range. We further hypothesize that the technique described above could be similarly applied to benefit other procedures.

## Introduction

Distal biceps tendon rupture occurs commonly in adult men [1], resulting from sudden eccentric contraction of the biceps brachii muscle [2]. Operative repair is the preferred treatment of distal biceps tendon rupture [3, 4]. Anatomical reinsertion with a one- or two-incision technique yields superior outcomes among multiple surgical techniques [5-7], with single-incision techniques rising in popularity in an effort to reduce post-operative complications [8, 9]. For instance, the cortical button is a commonly-used technique that provides robust strength with a single anterior incision [10-12].

The cortical button repair strategy carries a risk of posterior interosseous nerve (PIN) injury, which occurs with an estimated frequency of 3% [13, 14]. This repair is performed by drilling a tunnel through the radius, starting at the radial tuberosity and exiting dorsally. The metal cortical button is sutured to the distal portion of the biceps brachii and pulled through the tunnel. The button is then flipped on the dorsal aspect of the radius to affix the tendon on the bone. Theoretically, the PIN could be damaged by entrapment with the implant on the dorsal cortex of the proximal radius [15]. This damage may result in transient PIN palsy, and is an uncommon, but severe complication of distal biceps tendon rupture repair [16, 17].

Precise drill orientation through the radial tuberosity is important in avoiding PIN injury [13]. In order to fully visualize the radial tuberosity and achieve optimal drill angle and position, it is helpful for the surgeon to place the initial surgical incision as close as possible to the center of the tuberosity. Thus, the aim of this study was to create a set of equations or rules to guide the surgeon in making the optimal anterior incision for the procedure. Optimizing incision placement could then possibly make it easier to correctly orient the drill and to avoid PIN injury. We hypothesized that the olecranon tip, a palpable bony landmark, could be used as a reference point to estimate distance to the radial tuberosity center. We aimed to investigate how the distance from the olecranon tip to the radial tuberosity could be reliably estimated from patient demographic information.

## Materials and methods

Patient selection

All elbow X-rays dating between 2013 and 2015 at a single institution were reviewed. Patients were included in the study if at least two views (anteroposterior and lateral) were present. Patients were excluded based on the following criteria: a) prior surgery for distal biceps tendon repair; b) prior extensive surgery introducing any modifications or implants, including but not limited to full elbow arthroplasty; c) evidence of any fracture or bony deformity, malunion, or nonunion; d) evidence of severe degenerative change or osteoarthritis of the elbow joint. The 82 patients with plain film X-rays dated between 2013 and 2015 who satisfied the above criteria consisted of 54 men and 28 women with age ranging from 16 to 84 years (mean 48.2 years). Each of the 82 plain film X-ray series was confirmed to be from a distinct patient.

Radiographic analysis

**Figure 1:**
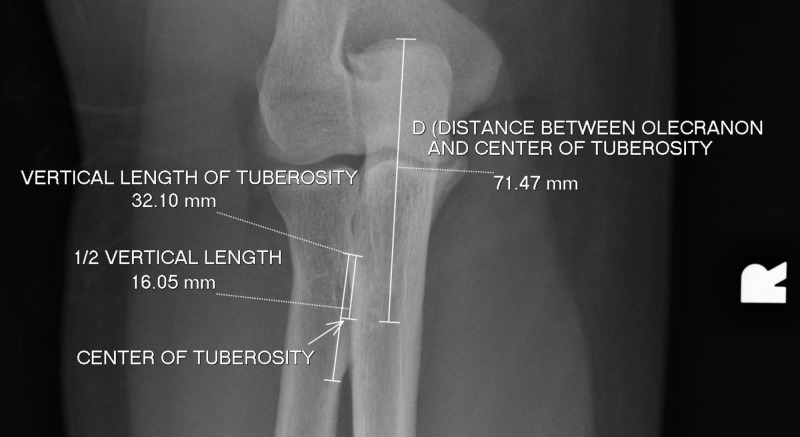
Measurement of olecranon to tuberosity distance. Radiographic measurement of the distance in mm between the olecranon tip and the center of the radial tuberosity using anteroposterior (AP) plain film X-rays.

Proximal and distal borders of the radial tuberosity were identified, and the center of the tuberosity was marked at the center of this length. Using anteroposterior (AP) views, the distance between the most proximal tip of the olecranon process and the center of the radial tuberosity (D) was measured (Figure 1).

If this was possible on more than one AP view, the mean of these measurements was recorded. Multivariate linear regression was used to relate this distance to age, sex, height, and body mass index (BMI).

Cadaveric study

Five cadaveric arms that had been harvested from three patients (Table 1) at mid-humerus and fully intact distally were obtained.

**Table 1:** Cadavers' clinical characteristics and surgical wire model validation. Five cadaveric arms from three patients were used to validate the statistical model predicted by radiographical measurements. The predicted distance ranged from 60.2 mm to 70.7 mm. The mean error (distance between surgical wire and center of tuberosity) was 1.8 mm. BMI: Body mass index.

Cadaver	Age	Height (m)	Weight (kg)	BMI	Sex	Arm	Predicted distance (mm)	Wire placement error (mm)
1	29	1.60	50.0	19.5	F	R	60.2	2
1	29	1.60	50.0	19.5	F	L	60.2	5
2	68	1.65	63.6	23.4	M	R	65.7	2
2	68	1.65	63.6	23.4	M	L	65.7	0
3	79	1.78	81.7	25.8	M	L	70.7	2

Donors’ sex, height, and BMI were used to calculate the expected olecranon tip to biceps tuberosity center length using the multivariate model equations. This distance was marked along the posterior aspect of the elbow held at 90° flexion and maximum supination. A 16 mm-diameter surgical wire was driven vertically through the proximal radius with the forearm in maximum supination at the marked site. A transverse volar incision was made in each specimen, centered over the guide pin at the location marked previously. Dissection was carried down toward the radial tuberosity. The distal tendon of the biceps was identified on each specimen, and its insertion onto the radial tuberosity was visualized. The error between the surgical wire and the center of tendon insertion was measured.

## Results

**Figure 2:**
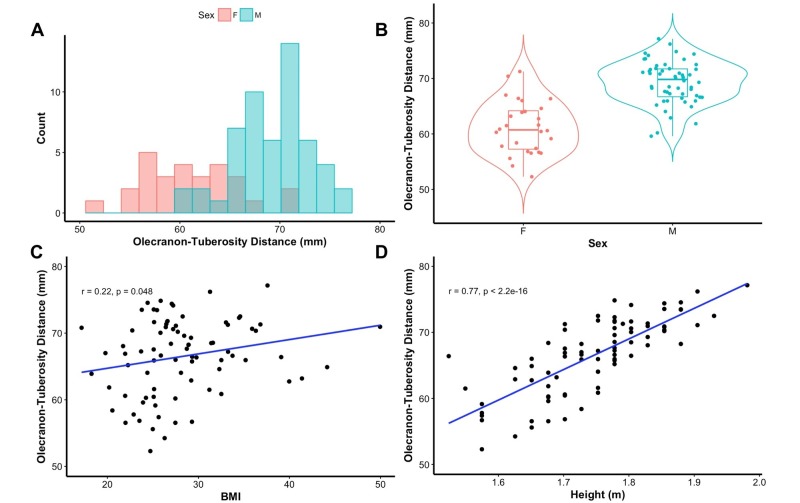
Association between clinical factors and olecranon-tuberosity distance. A) Histogram of distance measured radiographically between the olecranon tip and the center of the radial tuberosity. B) Sex was significantly associated with olecranon-tuberosity distance by univariate t-test (p < 0.001), with males having an average length of 69 mm, and females 61 mm. C) Body mass index (BMI) significantly correlated with distance (p < 0.05). D) Height significantly correlated with distance (p < 0.001).

The distance between the olecranon tip and the center of the radial tuberosity ranged from 52.3 mm to 77.2 mm (mean 66.5 mm) (Figure 2A). Males had significantly longer olecranon-tuberosity distance at a mean 69.3 mm compared to females, who had mean distance of 61.2 mm (t-test, p = 5.9 x 10^-10^) (Figure 2B). Increased BMI weakly correlated with increased distance (Pearson’s r = 0.22, p = 0.048). Height showed strong positive correlation with increased distance (r = 0.77, p < 0.001) (Figure 2C, 2D).

Multivariate regression revealed that significant predictive factors for olecranon-tuberosity distance were height (coefficient = 35.8, p < 0.001), BMI (coefficient = 0.14, p = 0.032), and male sex (coefficient = 3.17, p = 0.0039). The overall significance of the model was p < 0.001, with R2 value of 0.6685, suggesting that 67% of variance is accounted for by the model.

Using the coefficients of the multivariate regression model, we developed an algebraic formula to predict the olecranon-tuberosity distance (OTD):

$\ OTD= 35.9*(Height) + 0.14*(BMI) + 3.17*Male_{yes=1}$

By model predictions, we estimated the approximate tuberosity location relative to the olecranon tip in the cadaveric arms. The error (in mm) between the surgical wire placement and the center of the tuberosity in the cadaveric study ranged from 0 mm to 5 mm (mean 1.8 mm) (Table 1).

## Discussion

While prior studies have investigated the distance from the biceps tendon insertion on the radial tuberosity [18-21] and to estimate the location of the PIN [22, 23], this is the first study to our knowledge using the olecranon tip as a landmark. The results of our univariate and multivariate analyses suggest that distance from the olecranon tip of the elbow to the center of the bicipital tuberosity (OTD) significantly depends on height, BMI, and sex. The positive correlations of OTD to height and BMI are logical given anatomical proportionality of different individuals. Furthermore, our finding that males had a longer olecranon-tuberosity distance of 69.3 mm compared to females at 61.2 mm is consistent with prior studies. For instance, men were found to have a longer distance between the radial head and the biceps tuberosity (20.9 mm) compared to women (17.7 mm) [21]. Cadaveric study suggested that the model performed with 2 mm accuracy.

**Figure 3:**
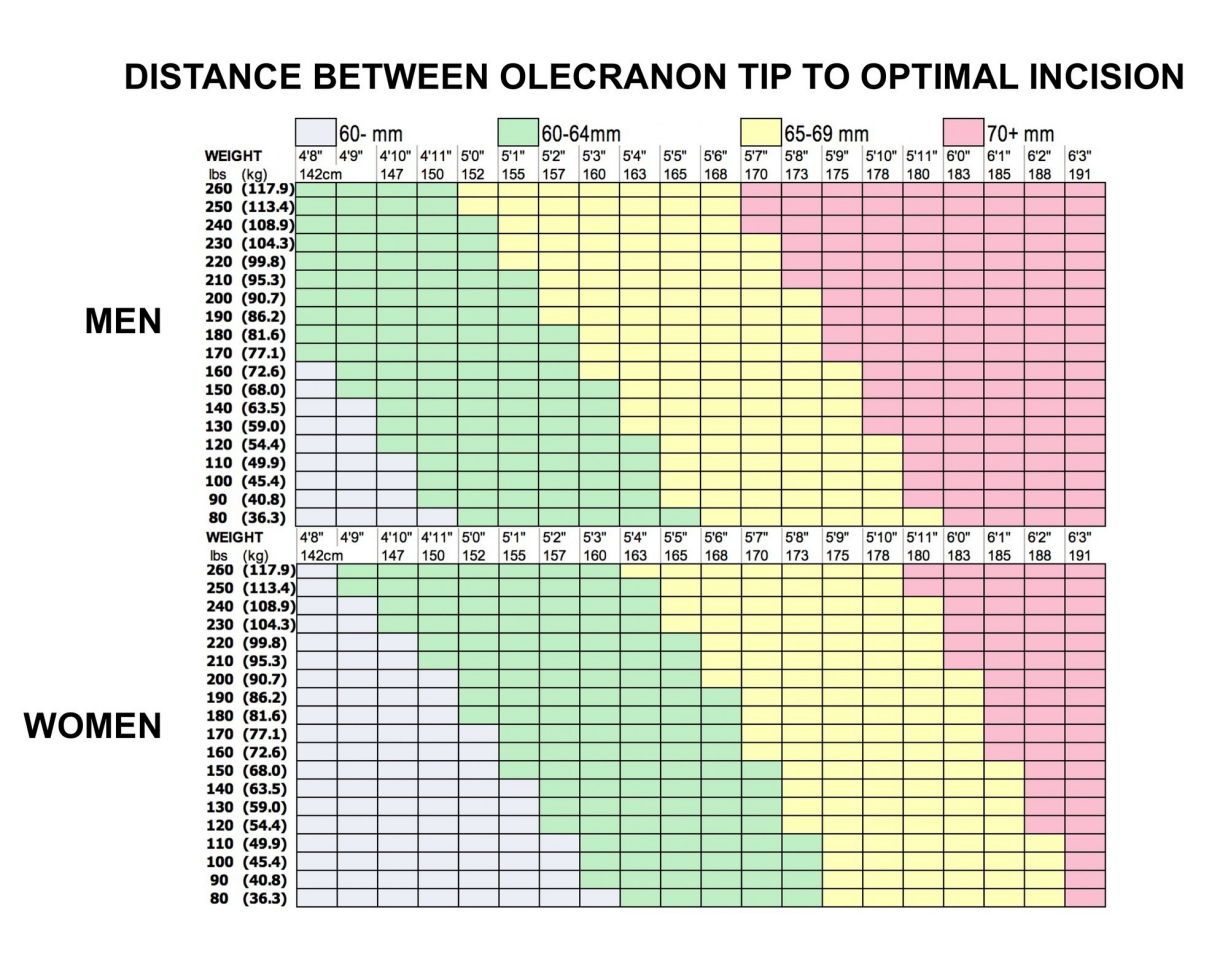
Optimal biceps tendon surgical incision placement rules. Guides for estimation of optimal volar incision placement based on sex, height, and weight.

The PIN is at risk of damage during a cortical button repair, as the repair is performed blindly without a second incision dorsally to confirm no nerve entrapment. We have used radiographic modeling of elbow anatomy in the hopes of guiding surgical incision placement. Using the multivariate regression model, we propose two tables, for male and female patients, as easily-accessible guides for orthopedic surgeons (Figure 3).

This study had several limitations. First, the cadaveric study focused on a small number of samples, and thus there were few data points for model validation. Second, the baseline variation between samples was within 20 mm, which is a narrow range. During surgery, a dissection is usually performed first to fully visualize the area, and thus the biceps tuberosity is likely to be visualized in its entirety even if the initial incision was optimized. Finally, this study did not prospectively evaluate whether implementation of this technique changes the rate of PIN injury. Given that the already low rate of PIN injury of approximately 3%, it is possible that minor adjustments to incision placement using the proposed technique may not significantly decrease PIN injury risk. Future prospective studies comparing PIN injury in cortical button repairs with and without incision placement optimization may be useful in validating the proposed optimization effort.

## Conclusions

PIN damage is a known complication of single-incision biceps tendon rupture repair with the cortical button technique. This study investigated the use of patient demographic factors to predict the distance between the olecranon tip of the elbow and the center of the radial tuberosity. Thus, using such a model, the olecranon tip can be used as a surgical landmark to estimate the preferred location for incision placement. The model was validated with surgical wire placement in five cadaveric arms and achieved accuracy within 2 mm. We propose that a radiographic modeling technique could be similarly applied to the study of other orthopedic procedures.
